# A three-month physical training program improves cardiovascular autonomic function in patients with metabolic syndrome with and without diabetes – a pilot study

**DOI:** 10.3389/fendo.2023.1224353

**Published:** 2023-08-17

**Authors:** Anna Vágvölgyi, Judit Erzsébet Ábrahám, Éva Máthéné Köteles, Andrea Korom, Mária Barnai, Mónika Szűcs, Andrea Orosz, Péter Kempler, Adrienn Menyhárt, Attila Nemes, Tamás Várkonyi, István Baczkó, István Kósa, Csaba Lengyel

**Affiliations:** ^1^ Department of Medicine, Albert Szent-Györgyi Medical School, University of Szeged, Szeged, Hungary; ^2^ Department of Medical Prevention, Albert Szent-Györgyi Medical School, University of Szeged, Szeged, Hungary; ^3^ Department of Physiotherapy, Faculty of Health Sciences and Social Studies, University of Szeged, Szeged, Hungary; ^4^ Department of Medical Physics and Informatics, Albert Szent-Györgyi Medical School, University of Szeged, Szeged, Hungary; ^5^ Department of Pharmacology and Pharmacotherapy, Albert Szent-Györgyi Medical School, University of Szeged, Szeged, Hungary; ^6^ Department of Oncology and Internal Medicine, Semmelweis University, Budapest, Hungary

**Keywords:** autonomic function, cardiovascular, diabetes, metabolic syndrome, physical exercise

## Abstract

**Introduction:**

Vascular complications and neuropathy may develop in the presence of metabolic syndrome. The aim of our study was to measure the cardiovascular autonomic function following physical training in patients with metabolic syndrome with and without diabetes.

**Subjects and methods:**

56 patients with metabolic syndrome (32 men/24 women, 40 non-diabetic patients (NDMetS)/16 diabetic patients (DMetS) [mean ± SD]: age: 50.35 ± 8.03 vs. 56.8 ± 9.30 years, p=0.023; baseline BMI: 32.2 ± 7.03 vs. 32.8 ± 5.94 kg/m^2^, p=0.739) were involved in our study. All tests and measurements were carried out before and following a 3-month physical training period. Autonomic function was assessed by means of five standard cardiovascular reflex tests. ECG repolarization parameters, including short-term QT variability and stress-ECG were also measured.

**Results:**

In the whole population, Valsalva-ratio (VR) and the autonomic score (AS) improved following training (VR: 1.49 ± 0.24 vs. 1.64 ± 0.34, p=0.001; AS: 2.05 ± 1.73 vs. 1.41 ± 1.36, p=0.015) accompanied by the significant decrease of the systolic (150.3 ± 16.12 vs. 134.1 ± 16.67 mmHg, p<0.001) and diastolic (90.64 ± 12.8 vs. 82.79 ± 11.1 mmHg, p<0.001) blood pressure. An improvement in VR was detected in NDMetS patients following training (1.51 ± 0.24 vs. 1.67 ± 0.31, p= 0.002). No significant changes could be detected in autonomic tests’ results in the DMetS patient group following training. The applied exercise training program did not lead to significant changes in ECG repolarization. The stress-ECG test in the whole study population yielded a significant increase in the test duration (12.9 ± 3.76 vs. 15.1 ± 2.96 min, p<0.001) and in the test load (10.5 ± 2.78 vs. 11.6 ± 2.39 MET, p<0.001). The load capability improved significantly in both subgroups: 11.1 ± 2.04 vs. 12.1 ± 1.82, (p<0.001) and 9.0 ± 3.64 vs. 10.4 ± 3.05, (p=0.033) in subpopulations of NDMetS and DMetS, respectively. The DMetS patients achieved a significantly lower MET score at baseline (p=0.039) and following training (p=0.044) in comparison to the NDMetS patients.

**Conclusion:**

The three-month exercise program improved the Valsalva-ratio and the AN score in the MetS patients, that is potentially protective against cardiovascular events. The training had some beneficial effect on blood pressure and the results of the stress-ECG tests in both groups. The absence of significant change in the reflex tests in DMetS group reflects an impaired adaptation compared to the NDMestS group.

## Introduction

Obesity and its related consequences are associated with a significant health risk worldwide, and its management represents a huge financial burden both in the form of direct health care costs and social expenditures. Metabolic syndrome (MetS), a co-occurrence of abdominal obesity, dyslipidemia (elevated triglyceride levels and reduced high-density lipoprotein cholesterol) (1), hyperglycemia and hypertension, poses a major public health challenge. The current criteria for the diagnosis of metabolic syndrome include meeting at least three out of five previously mentioned conditions, which leads to a heterogeneity of the population of patients diagnosed with MetS. Among people with MetS might be present three to five components of metabolic syndrome, while there are patients with MetS with the same number, but different components of criteria for MetS. Jakubiak et al. have found that obesity and insulin resistance have been shown to be the component that contributes the most to the relationship between the metabolic syndrome and oxidative stress (2). Metabolic syndrome is expected to increase cardiovascular mortality by 1.8 times in the next 15 years (3), while another study predicts a two to fivefold increase in the risk of developing cardiovascular diseases and type 2 diabetes mellitus (T2DM) in the next 5 to 10 years (1). In developed countries, MetS affects around one fifth to one fourth of the population (4–6). In the prevention and treatment of MetS, lifestyle changes, especially regular physical exercise are recommended. In addition to aiming at the slowing and reversal of obesity-related pathological conditions, recently there has been an increased emphasis on the implementation of preventive measures.

The International Diabetes Federation (IDF) positions lifestyle intervention at the forefront of the treatment of metabolic syndrome as a primary intervention (7). Corresponding to this, IDF aims to increase physical activity, change dietary habits and achieve a 5-10% reduction in body weight. Pharmacotherapy to reduce dyslipidemia, hypertension or insulin resistance should only be considered as a secondary intervention in cases where lifestyle interventions have failed (6).

Cardiovascular autonomic neuropathy (CAN) is defined as impaired autonomic control of the cardiovascular system, after the exclusion of other causes. CAN is a major complication of type 1 and type 2 diabetes mellitus; however, recent data suggest that CAN also occurs in obesity (8) and it is increasingly recognised in people with prediabetes and MetS, with a reported prevalence of up to 11% and 24%, respectively (9). CAN is associated with increased cardiovascular morbidity and the diagnosis of CAN is associated with a relative risk of mortality increase of at least 3.65 (10). CAN promotes ventricular repolarization disturbances that manifest as heart rate-corrected QT interval (QTc) prolongation and increased QT dispersion (QTd) on the ECG, both suggesting increased myocardial electrical instability, a substrate for fatal ventricular arrhythmias (11, 12).

Autonomic dysfunction and MetS in obese patients have been associated with normal glucose tolerance (NGT) and an increased prevalence of cardiovascular disease (13). A significant association has been shown between higher body mass index (BMI) and an elevated risk of CAN (14). In a randomized controlled trial, CAN was found to be associated with central obesity and impaired glucose tolerance with a prevalence of parasympathetic dysfunction observed in 25% of patients and a prevalence of sympathetic dysfunction observed in only 6% of patients (15).

In the obese population, calorie-controlled diet combined with regular exercise-based lifestyle interventions are crucial to prevent CAN (9, 16–18). Physical activity alone such as walking, moderate endurance and aerobic exercise demonstrated improvements in the cardiac autonomic function including heart-rate variability (19). Kluding et al. (20) published the first study, which described improvements in cutaneous nerve fiber branching following a supervised 10-week aerobic and strengthening exercise program in people with diabetic peripheral neuropathy, in 2012. Furthermore, in T2DM patients, a significant improvement was detected in heart-rate variability following a 6-month long aerobic exercise-training program (21).

Máthéné Köteles et al. in 2023 (22) studied the physiological and psychological effects of a 12-week home-based and telemonitored physical exercise program in patients with metabolic syndrome. They involved 55 MetS patients who were instructed to perform 3–5 sessions of physical activity (at least 150 min) each week. Trainings were monitored off-line by heart rate sensors (smart watch or chest strap), and the data were transferred to an online platform by a fitness application through a cloud-based data transfer system, where physiotherapists supervised the training sessions giving weekly feedback to patients. The study revealed significant changes in several parameters, including waist and hip circumferences, 6-minute walking distance, maximal exercise capacity in METS, stress electrocardiogram duration time, body weight, and certain laboratory parameters such as HDL-cholesterol, fasting plasma glucose, and glycated hemoglobin A1c. Additionally, a notable reduction in the overall scores of the Maastricht Vital Exhaustion Questionnaire and an increase in the overall scores of the WHO Wellbeing Scale were also observed. They concluded that a 12-week home-based telemonitored training supported by an affordable and commonly available telemonitoring system could produce positive, statistically significant changes in many physical and psychological components in MetS patients.

There is little data in the literature regarding the effect of physical exercise on cardiovascular autonomic function and ECG parameters in patients with metabolic syndrome. Therefore, in the present study, we investigated whether our three-month-long home based exercise intervention program, supervised by remote patient monitoring, can improve cardiovascular autonomic nerve function. We also studied whether the training program had any effect on different ECG parameters that would suggest beneficial changes in repolarization instability, a known substrate for severe ventricular arrhythmias.

## Subjects and methods

### Subjects

#### Inclusion criteria

Metabolic syndrome is defined by the National Cholesterol Education Program Adult Treatment Panel III (NCEP ATP III) MetS criteria (1, 23). Voluntary patients, aged between 25 and 70 years, were involved in the study, who practiced only low level of regular physical activity (self-reported, less than 30 min a week), and had at least three risk factors concomitantly from the followings (1, 23):

- waist circumference above 102 cm in men and above 88 cm in women,
- proved type 2 diabetes mellitus or FPG level above 5.6 mmol/L,
- treated hypertension or spontaneous blood pressure above 130/85 mmHg,
- treated hypertriglyceridemia or serum triglyceride level above 1.7 mmol/L,
- serum HDL (high-density lipoprotein) cholesterol level under 1.03 mmol/L in men, under 1.3 mmol/L in women.

#### Exclusion criteria

Participants were excluded with: any upcoming planned invasive cardiovascular intervention (percutaneous transluminal coronary angioplasty, coronary artery bypass, valve repair or replacement), chronic heart failure, uncontrolled hypertension (blood pressure > 160/100 mmHg), type 1 diabetes mellitus (T1DM), T2DM which needed more than one dose of insulin per day, chronic renal failure where the estimated Glomerular Filtration Rate < 60 ml/min/1.73 m^2^, oncological diseases, serious cognitive dysfunction, lack of cooperation, any known disease or condition that seriously affected the mental and legal capacity, any other conditions preventing regular physical trainings.

The present study involved 56 patients diagnosed with metabolic syndrome [32 men/24 women, 40 non-diabetics (NDMetS)/16 diabetics (DMetS) {mean ± SD}: age: 50.35 ± 8.03 vs. 56.8 ± 9.30 years, p=0.023; baseline BMI: 32.2 ± 7.03 vs. 32.8 ± 5.94 kg/m^2^, p=0.739]. Patients were monitored at the Department of Preventive Medicine, University of Szeged, Szeged, Hungary. The diabetic group consisted of patients with previously known diabetes or with HbA1c% above 6.5% in the baseline laboratory measurements.

All of the participants were of Caucasian origin. The relevant clinical data of MetS patients are shown in **Table 1**. Patients with NDMetS were significantly younger (50.4 ± 8.03 vs. 56.8 ± 9.30 years, p=0.023), presented with an elevated resting diastolic blood pressure (93.1 ± 13.54 vs. 84.6 ± 8.36 mmHg, P=0.007) and significantly fewer of them suffered from hypercholesterinemia as comorbidity (7 vs. 10, P=0.03). According to further biometrical parameters and medication-taking pattern, only anticoagulants and antidiabetics except insulin intake were significantly higher in the DMetS group. The medication of the subjects did not change during the examination period.

**Table 1:** Relevant baseline clinical data in the study groups.

	Non-Diabetic MetS Patients (n=40)	Diabetic MetS Patients (n=16)	P-value
Clinical Data			
Age (year)	50.4 ± 8.03	56.8 ± 9.30	***0.023***
Body Mass Index (BMI)	32.17 ± 7.03	32.8 ± 5.94	0.739
Waist-hip-ratio (WHR)	0.92 ± 0.11	0.94 ± 0.09	0.601
Male sex (%)	22 (55)	10 (63)	0.767
Systolic BP (mmHg)	150.8 ± 16.76	149.1 ± 14.8	0.724
Diastolic BP (mmHg)	93.1 ± 13.54	84.6 ± 8.36	***0.007***
Heart Rate (1/min)	73.44 ± 12.31	71.56 ± 10.96	0.583
Smoking history (%)	13 (33)	9 (56)	0.134
Alcohol consumption (%)	14 (35)	3 (19)	0.339
Hypertension (%)	25 (63)	11 (69)	0.763
Hypercholesterolemia (%)	7 (18)	10 (63)	***0.003***
Medication			
β-blocker (%)	11 (28)	7 (44)	0.343
ACE inhibitor or ARB (%)	20 (50)	11 (69)	0.245
Ca-antagonist (%)	9 (23)	7 (44)	0.189
Antidiabetics except insulin (%)	0 (0)	10 (63)	***<0.001***
Insulin (%)	0 (0)	2 (13)	0.078
Statin (%)	7 (18)	4 (25)	0.711
Diuretics (%)	15 (38)	6 (38)	0.999
ASA/PAI (%)	11 (28)	2 (13)	0.308
Anticoagulants	1 (3)	4 (25)	***0.020***

P-values below 0.05 level are marked bold. Values are presented as mean ± SD. BP, blood pressure; ACE, angiotensin-converting enzyme; ARB, angiotensin-receptor blocker; Ca, calcium; ASA, acetylsalicylic acid; PAI, platelet aggregation inhibitor.

### Intervention

Following the assessments, the participants were informed about the details and features of the exercise program and the monitoring procedures. They were requested to perform 3-5 training sessions (minimum 30 minutes/session), for a minimum of 150 minutes each week at home, for a total of 12 weeks. There were no restrictions regarding the type of training they performed, but patients were educated on the types and intensities of training recommended for individuals with metabolic syndrome, as outlined in the existing literature (24–26). The range of the training intensity was calculated based on the age dependent maximal heart rate (60-80%). Patients were also educated on the application of the chosen training monitor device and the procedures of the data transfer.

#### Home-based training sessions with remote patient monitoring

As many as 42 patients (10 with DMestS) participated in the home-based training sessions, which were monitored by two different types of devices. One of them was an electric heart rate sensor (Polar H10, Kempele, Finland) with a chest strap. Data collection was performed using a freely downloadable fitness application (Polar Beat) on a connected smartphone provided for the participants. At the end of the training session, the application automatically synchronized and transferred the data to a cloud based integrated system for online coaching (https://flow.polar.com/coach). The other type of device used was a smart watch with optical heart rate sensor (Polar M430 GPS running watch, Kempele, Finland). This watch was capable of initiating and stopping the training sessions. However, the data synchronization required, a patient-owned smart phone running the free to download Polar Flow fitness application or a patient-owned PC running the Polar Flow website. In this setting it was the patients’ responsibility to initiate regular data upload using wireless connection of the smart phone or wired connection of the PC. The patients performed their exercise program in their home environment while the physiotherapists provided offline monitoring and coaching for the training sessions, weekly contacting the patients *via* phone or email.

#### Supervised ambulatory training sessions

Fourteen patients (6 with DMestS) participated in the ambulatory supervised training group. These patients were scheduled for a 45-60-minute ambulatory training twice a week as well as a self-managed training session in their homes once a week. The ambulatory training sessions were supervised by physiotherapists. One of the two supervised training sessions was mainly a dynamic, endurance type training and consisted of a warm up period, followed by either ergometer cycling, treadmill run, or elliptical training and a cool down stage, altogether covering 60 minutes. The other session consisted of a resistance circle training including a warm up period, combined leg and arm exercises using the patients’ own body weight, dumbbells and a cool down stage, also lasting for 60 minutes. During the supervised ambulatory training sessions, the subjects’ heart rate was continuously monitored with a chest strap Polar Team2 device (Kempele, Finland). This system allowed for simultaneous online monitoring of the heart rate of every person in the group, so the physiotherapist could continuously follow the whole group during the sessions. The third training of the week consisted of a home-based training session, self-managed and self-reported by the participants, and of a minimum 30-minute-long activity of their own choice.

### Assessments

#### Ewing’s five standard cardiovascular reflex tests, autonomic score

Autonomic neuropathy (AN), neuronal dysfunction and consequent cardiovascular changes were characterized with Ewing’s five standard cardiovascular reflex tests (27) in the study. The Ewing-tests are the gold standards for diagnosing autonomic dysfunction; they provide non-invasive, clinically relevant, standardised and reproducible data of autonomic functions. Reflex tests were performed by measuring the blood pressure and obtaining continuous 6-lead ECG signals. The signals were digitised with a multichannel data acquisition system (Cardiosys-A01 software, MDE Heidelberg GMBH, Heidelberg, Germany), the sampling rate was 2 kHz and the data were stored for later analysis.

Three of these tests record heart rate changes when performing specific activities, while the rest measures changes in blood pressure. Tests, which record heart rate changes predominantly, reflect changes in parasympathetic function, while those based on blood pressure responses primarily describe sympathetic function disturbances (28). Heart rate changes were measured during deep inhalation and exhalation, in lying and standing positions with 30/15 ratio, and during and after Valsalva manoeuvre (10). Systolic blood pressure changes were measured after standing up from a lying position, while diastolic changes were recorded during gripping with the hand for 3 minutes.

CRT-s were scored separately: 0 (normal), 1 (borderline), 2 (abnormal). The overall autonomic score was calculated from the sum of each test result to characterise the severity of AN.

#### Heart rate response to deep breathing

Physiologically the heart rate increases on inhalation and decreases on exhalation. Patients were instructed to take deep breaths at a rate of six breaths per minute (inhale for five seconds and exhale for five seconds). The difference between the measured maximum and minimum heart rates (beats/min) was calculated during six cycles of breathing.

#### Heart rate response to standing up (30/15 ratio)

Normally, the heart rate increases promptly after standing up from a lying position and at about the 15^th^ heartbeat after standing up, it reaches a peak. After that, relative bradycardia presents in healthy individuals with the lowest rate at around the 30^th^ beat. Patients were lying in a supine position at the beginning of the test then they were asked to stand up while the ECG was recorded continuously. The ratio of the longest R-R interval (around beat 30^th^) and the shortest R-R interval (around beat 15^th^) was calculated and recorded as the 30/15 ratio.

#### Heart rate response to the Valsalva manoeuvre (Valsalva-ratio)

In healthy individuals the blood pressure decreases and the heart rate increases during Valsalva manoeuvre. After the manoeuvre, the blood pressure increases and the heart rate decreases. Subjects were asked to exhale into a specific manometer through a mouthpiece and hold their breath at 40 mmHg for 15 seconds. During that period of time, ECG was continuously recorded. The ratio of the longest R-R interval following the test and the shortest R-R interval during the manoeuvre was calculated and recorded as the Valsalva-ratio.

#### Systolic blood pressure response to positional change (from lying to standing up)

Normally, in a standing position, the redistribution of blood to the lower limbs is immediately compensated by vasoconstriction in the peripheral vessels. Marked orthostatic hypotension is an important feature of cardiovascular consequences of neuropathy. The test is performed by measuring the blood pressure in a lying position and after standing up. An orthostatic drop in blood pressure is determined with systolic blood pressure measurements at 10 minutes after lying supine and at 1, 5 and 10 minutes after standing up. The difference between the values measured is noted and the largest difference is recorded as the response to standing up.

#### Diastolic blood pressure response during sustained handgrip

Changes in diastolic blood pressure were measured during sustained handgrip. At first, patients were asked to clamp a hand-held dynamometer with their dominant hand exerting full force so that we could determine the maximal grasping force, then they were instructed to maintain the grasp for 3 minutes at a constant, 30% force level. Blood pressure was measured once every minute on the contralateral, relaxed upper limb and the maximally increased diastolic blood pressure was recorded as the response to sustained handgrip.

#### 12-lead electrocardiogram

All patients before and after the training exercise intervention were examined in the supine position following 5 minutes of rest. The 12-lead ECG was performed continuously for 10 minutes in the same position to avoid motion artefacts as much as possible. The ECG signal digitisation was performed with a multichannel data acquisition system (CAR03-IA, Cardiosys EXTRA software, MDE Heidelberg GmbH, Heidelberg, Germany), the sampling rate was 2 kHz and the data were stored for later analysis. All data acquired from subjects were appropriate for analysis. No data were excluded on the basis of exclusion criteria, which were the following: implanted pacemaker, several (>5%) ectopic atrial or ventricular beats, non-sinus rhythm, abnormal repolarization (i.e. early repolarization, T-wave inversion, complete bundle branch block), acute metabolic disorder, significant artefacts in the ECG signal recording, high amount of food intake during the last 3 hours, alcohol or caffeine consumption or smoking during the last 10 hours. Repolarization was analysed based on the following parameters: frequency corrected QT interval (QTc) using Bazett (QTc = QT/√RR), Fridericia (QTc = QT/[RR/1,000]1/3), Framingham (QTc = QT + [0.154 × {1,000 − RR}]) and Hodges formulae (QTc = QT + 1.75 × [60,000/RR − 60]), QT dispersion (QTd), terminal T-wave duration (T_peak_ - T_end_), frequency corrected T_peak_ - T_end_ interval using Bazett and Fridericia formulae and short-term variability of QT intervals (STV_QT_). The RR and QT intervals, as well as the duration of the T wave from the peak to the end (T_peak_ - T_end_) intervals were measured semi-automatically from 30 consecutive beats (minimum number of intervals needed for variability measurements) and were calculated as the average of 30 beats. Conventional computerised QT measurement technique was used for the analysis of QT intervals; blinded QT interval checking and if necessary, manual repositioning of the automatically set fiducial cursor were performed by the same investigator of the team (29). The duration of QTc interval was determined as the average of the measured QTc intervals. PQ and QRS intervals were determined as the average of the measured intervals of 15 consecutive beats. The measurements were performed in lead II or lead V5 if significant noise was present in the former. Poincaré plot analysis of the QT and RR intervals was performed to determine the temporal instability of the beat-to-beat heart rate (HR) and repolarization. Each QT and RR value was plotted against its former value. STV_QT_ and STV_RR_ were calculated using the following formula: STV = Σ|D_n+1_ − D_n_| (30x√2)^−1^, where D represents the duration of the QT and RR intervals. The calculation defines the STV as the mean distance of points perpendicular to the line of identity in the Poincaré plot and relies on previous mathematical analysis (30).

#### Stress-electrocardiogram

Under the supervision of a cardiologist, a 12-channel electrocardiogram (CardioSys, MDE Diagnostic, Walldorf, Germany) was performed at rest and during exercise using an incremental loading in accordance with the Modified Bruce Protocol until the age-predicted maximal heart rate, where this target heart rate was calculated based on the 220-age formula. The maximal capacity in Metabolic Equivalent of Task (MET; ml/kg/min), the maximal heart rate (bpm), maximal systolic and diastolic blood pressure (mmHg) and the duration of stress-electrocardiogram test in minutes were measured and documented.

### Statistical analysis

Data are reported as the mean ± SD; or as frequencies (n) and percentages (%), when appropriate. Independent samples t-test and Fisher exact test were used for the comparison the baseline clinical data in the study groups. Repeated measures variance analysis (RM-ANOVA) was applied for the analysis cardiovascular autonomic function tests (**Table 2**) and of ECG parameters (**Table 3**) before and after physical training where the grouped variables were the diabetic and non-diabetic subgroups. For pairwise comparisons, Bonferroni correction was used. Statistical tests were performed using R statistical software (R version 3.6.1), values of p<0.05 were considered significant. Prior power analysis using the statistical software G-Power (Version 3.1.9.7) calculated an optimal total sample size of N=52, with an effect size f=0.3, alpha as Type I error of 0.05 and a power of 0.85.

**Table 2:** Results of the cardiovascular autonomic function tests before and following the 3-month training program in all patients (n=56) and with sub-analyzation in non-diabetic (NDMetS; n=40) and in diabetic (DMetS; n=16) patients.

Cardiovascular Reflex Tests	Before training all patients (n=56)	Following training all patients (n=56)	Before Training NDMetS (n=40)	Following Training NDMetS (n=40)	Before Training DMetS (n=16)	Following Training DMetS (n=16)	P-value All patients before vs. following training	P-value NDMetS before vs. following training	P-value DMetS before vs. following training	P-value 1.M. DMetS vs. NDMetS	P-value 2.M. DMetS vs. NDMetS
**HRRDB (1/min)**	20.6 ± 8.17	22.3 ± 8.52	22.1 ± 8.56	23.1 ± 8.64	17.1 ± 6.01	20.3 ± 8.13	0.109	0.407	0.081	***0.019***	0.258
**VR**	1.5 ± 0.24	1.6 ± 0.34	1.5 ± 0.24	1.7 ± 0.31	1.4 ± 0.23	1.6 ± 0.40	***0.001***	***0.002***	0.189	0.336	0.411
**HRRSU**	1.1 ± 0.13	1.1 ± 0.12	1.1 ± 0.12	1.1 ± 0.13	1.2 ± 0.15	1.2 ± 0.10	0.698	0.542	0.721	0.256	0.504
**SBPRSU (mmHg)**	6.9 ± 8.57	4.9 ± 8.10	6.4 ± 7.13	4.4 ± 7.48	8.4 ± 11.57	6.2 ± 9.61	***0.055***	0.126	0.259	0.509	0.506
**DBPRH (mmHg)**	17.3 ± 16.06	17.8 ± 11.77	18.2 ± 16.81	17.9 ± 12.40	14.8 ± 14.22	17.6 ± 10.37	0.824	0.903	0.432	0.448	0.945
**Autonomic score**	2.1 ± 1.73	1.4 ± 1.36	1.9 ± 1.58	1.4 ± 1.37	2.6 ± 2.03	1.5 ± 1.37	***0.015***	0.11	0.059	0.221	0.76
**Systolic BP (mmHg)**	150.3 ± 16.12	134.1 ± 16.67	152.0 ± 16.56	132.7 ± 16.82	146.1 ± 14.62	137.7 ± 16.26	***<0.001***	***<0.001***	***0.024***	0.198	± 0.313
**Diastolic BP (mmHg)**	90.6 ± 12.80	82.8 ± 11.1	91.4 ± 13.28	83.2 ± 11.14	88.8 ± 11.68	81.8 ± 11.3	***<0.001***	***0.001***	***0.002***	0.467	0.685

P-values below 0.05 level are marked bold. Values are presented as mean ± SD. 1.M, first measurement; 2.M, second measurement; HRRDB, the heart rate response to deep breathing; VR (Valsalva-ratio), the heart rate responses to Valsalva maneuver; HRRSU (30/15 ratio), the heart rate responses to standing up; SBPRSU, the systolic blood pressure response to standing up; DBPRH, the diastolic blood pressure response during sustained handgrip; BP, blood pressure.

**Table 3:** ECG parameters of all patients (n=56) and subgroups before and following the 3-month training program in diabetic (DMetS; n=16) and non-diabetic (NDMetS; n=40) patients evaluated by RM-ANOVA: pairwise comparisons with Bonferroni corrections.

ECG Parameters	Before training all patients (n=56)	Following training all patients (n=56)	Before Training NDMetS (n=40)	Following Training NDMetS (n=40)	Before Training DMetS (n=16)	Following Training DMetS (n=16)	P-value All patients before vs. following training	P-value NDMetS before vs. following training	P-value DMetS before vs. following training	P-value 1.M. DMetS vs. NDMetS	P-value 2.M. DMetS vs. NDMetS
**HR (1/min)**	72.7 ± 11.83	72.71 ± 9.91	73.5 ± 12.30	72.3 ± 8.77	70.8 ± 10.69	73.7 ± 12.58	0.999	0.481	0.104	0.412	0.696
**RR (ms)**	846.7 ± 137.40	842.6 ± 124.82	839.1 ± 140.06	843.6 ± 108.12	865.8 ± 132.94	840.3 ± 163.47	0.783	0.813	0.285	0.51	0.941
**QT (ms)**	401.5 ± 33.49	403.4 ± 33.40	398.3 ± 31.97	403.7 ± 30.59	409.6 ± 36.84	402.7 ± 40.72	0.555	0.187	0.129	0.295	0.927
**QTc Bazett (ms)**	438.2 ± 21.67	440.8 ± 22.54	436.9 ± 20.86	440.6 ± 23.37	441.6 ± 23.94	441.3 ± 21.04	0.246	0.178	0.952	0.500	0.912
**QTc Fridericia (ms)**	425.3 ± 21.11	427.8 ± 22.91	423.3 ± 19.44	427.8 ± 23.25	430.4 ± 24.79	427.7 ± 22.76	0.231	0.078	0.389	0.319	0.988
**QTc Framingham (ms)**	425.1 ± 20.09	427.7 ± 21.68	423.1 ± 18.23	427.8 ± 22.03	430.2 ± 24.02	427.3 ± 21.50	0.206	0.062	0.32	0.296	0.935
**QTc Hodges (ms)**	423.8 ± 20.90	425.5 ± 22.58	421.9 ± 18.83	425.1 ± 21.79	428.5 ± 25.43	426.5 ± 25.16	0.34	0.15	0.539	0.357	0.841
**PQ (ms)**	159.2 ± 19.83	160.9 ± 19.39	158.8 ± 21.82	159.0 ± 21.89	159.9 ± 15.01	165.1 ± 11.55	0.212	0.924	0.013	0.834	0.194
**QRS (ms)**	99.3 ± 16.08	99.3 ± 15.46	98.9 ± 16.52	98.6 ± 15.80	100.2 ± 15.43	100.9 ± 14.96	0.987	0.815	0.740	0.788	0.621
**T_peak_-T_end_ (ms)**	87.4 ± 16.05	87.1 ± 15.68	84.5 ± 15.69	87.4 ± 16.24	94.9 ± 14.91	86.3 ± 14.66	0.865	0.170	***0.043***	***0.027***	0.795
**T_peak_-T_end_ (ms) Bazett**	95.5 ± 16.18	95.0 ± 15.31	96.1 ± 17.34	95.7 ± 15.80	93.9 ± 13.31	93.2 ± 14.39	0.803	0.854	0.866	0.605	0.576
**T_peak_-T_end_ (ms) Fridericia**	92.7 ± 15,80	92.2 ± 15.18	93.2 ± 17.00	92.9 ± 15.87	91.3 ± 12.79	90.4 ± 13.68	0.813	0.896	0.820	0.657	0.570
**QTd**	38.3 ± 10.88	39.7 ± 15.32	38.0 ± 10.98	37.4 ± 12.33	39.1 ± 10.96	45.4 ± 20.39	0.477	0.764	0.212	0.734	0.156
**STV_RR_**	16.5 ± 18.72	15.01 ± 17.44	13.9 ± 12.56	14.1 ± 13.37	23.2 ± 28.43	17.3 ± 25.34	0.269	0.870	0.086	0.224	0.634
**STV_QT_**	4.6 ± 1.10	4.5 ± 1.33	4.5 ± 1.19	4.4 ± 1.10	4.7 ± 0.89	4.9 ± 1.78	0.883	0.520	0.637	0.625	0.348

P-values below 0.05 level are marked bold. Values are presented as mean ± SD. 1.M, first measurement; 2.M, second measurement; HR, heart rate; QTc, frequency corrected QT interval (calculated by the Bazett, Fridericia, Framingham and Hodges formulas); Tpeak-Tend, duration of the T wave from the peak to the end; Tpeak-Tend Bazett/Fridericia, frequency corrected Tpeak-Tend calculated by the Bazett, Fridericia formulas; QTd, QT dispersion; STVRR, beat-to-beat short-term temporal variability of the RR interval; STVQT, beat-to-beat short-term temporal variability of the QT interval.

## Results

The three-month training program was considered effective, which was demonstrated by a significant decrease of the BMI from 32.4 ± 6.67 kg/m^2^ to 31.8 ± 6.76 kg/m^2^ (p=0.008). The weekly training activity duration was 155.4 ± 113.5 min in the undivided population. There was no significant difference in the duration of the weekly training activity between the NDMetS and DMetS subgroups: 165.7 ± 117.95 min vs. 137.6 ± 94.91 min; p=0.412.

### Cardiovascular autonomic function tests before and following the 3-month training program

The results of the cardiovascular autonomic function tests before and following the 3-month training program are shown in **Table 2**. In the whole population VR and the autonomic score improved significantly following the training (VR: 1.49 ± 0.24 vs. 1.64 ± 0.34, p=0.001; AS: 2.05 ± 1.73 vs. 1.41 ± 1.36, p=0.015, respectively) accompanied by significant decreases in the resting systolic (150.3 ± 16.12 vs. 134.1 ± 16.67 mmHg, p<0.001) and diastolic (90.64 ± 12.8 vs. 82.79 ± 11.1 mmHg, p<0.001) blood pressures.

SBPRSU following the training in the whole population also showed an improvement tendency, but the degree of change did not reach the level of statistical significance (6.95 ± 8.57 vs. 4.89 ± 8.10 mmHg, p=0.055).

Considering the subgroups, a significant improvement in VR was detected in the NDMetS patients following training (1.51 ± 0.24 vs. 1.67 ± 0.31, p= 0.002), but no significant change could be detected in the autonomic test results in the DMetS patient group.

At baseline, the diabetic subpopulation demonstrated significantly impaired results on the HRRDB test in comparison to NDMetS patients: 17.12 ± 6.01 vs. 22.05 ± 8.56 (p=0.019). No further significant differences in the results of autonomic tests could be detected between the two subpopulations at either the baseline or the second measurement.

Similar to the entire population, there was a significant reduction in resting systolic and diastolic blood pressure (BP) in both the NDMetS (systolic BP: 152.0 ± 16.56 vs. 132.7 ± 16.82 mmHg, p<0.001; diastolic BP: 91.4 ± 13.28 vs. 83.2 ± 11.14 mmHg, p=0.001, respectively) and the DMetS groups (systolic BP: 146.1 ± 14.62 vs. 137.7 ± 16.26 mmHg; p=0.024; diastolic BP: 88.8 ± 11.68 vs. 81.8 ± 11.30 mmHg, p=0.002, respectively).

### ECG parameters before and following the 3-month training program

Comparing ECG parameters in all MetS patients revealed no significant differences in heart rate, the PQ, QRS and QT intervals and the QT dispersion. Furthermore, significant differences in the QTc values calculated with the Bazett, Fridericia, Framingham and Hodges formulae and the beat-to-beat short-term temporal variability of the QT interval were not identified (**Table 3**). The resting ECG parameters did not show any significant differences between the diabetic and non-diabetic subpopulations at baselie. After 3 months of training, significant changes were documented only in T_peak_ - T_end_ parameter in DMetS subjects before vs. following training (p=0.043) and following subanalysation of baseline values between the DMetS and NDMetS groups (p=0.027). As T_peak_ - T_end_ is heart rate dependent, a correction was applied with Bazett and with Fridericia formulae. The above mentioned significant differences in T_peak_ - T_end_ following frequency correction with Bazett and Fridericia formulae could not be verified.

### Stress-electrocardiogram parameters before and following the 3-month training program

The exercise capacity of patients, as indicated by the duration of the stress-electrocardiogram test and the load values performed, exhibited a significant increase in the entire study population (12.9 ± 3.76 vs. 15.1 ± 2.96 min, p<0.001 and 10.5 ± 2.78 vs. 11.6 ± 2.39 MET, p<0.001) (**Table 4**). The DMestS patients achieved a significantly lower MET score at baseline (p=0.039) as well as following the training (p=0.044) in comparison to the NDMetS patients. The increase of MET value was consequent both in the NDMetS (11.1 ± 2.04 vs. 12.1 ± 1.82, p<0.001) and in the DMetS (9.0 ± 3.64 vs. 10.4 ± 3.05, p=0.033) subpopulations.

**Table 4:** Stress-Electrocardiogram parameters of all patients (n=50) and subgroups before and following the 3-month training program in diabetic (DMetS; n=15) and non-diabetic (NDMetS; n=35).

Stress-Electrocardiogram Parameters	Before training all patients (n=50)	Following training all patients (n=50)	Before Training NDMetS (n=35)	Following Training NDMetS (n=35)	Before Training DMetS (n=15)	Following Training DMetS (n=15)	P-value All patients before vs. following training	P-value NDMetS before vs. following training	P-value DMetS before vs. following training	P-value 1.M. DMetS vs. NDMetS	P-value 2.M. DMetS vs. NDMetS
**Duration (min)**	12.9 ± 3.76	15.1 ± 2.96	13.3 ± 3.56	15.6 ± 2.51	11.9 ± 4.13	13.8 ± 3.56	***<0.001***	***<0.001***	***0.009***	0.231	0.083
**Maximal exercise capacity (MET score)**	10.5 ± 2.78	11.6 ± 2.39	11.1 ± 2.04	12.1 ± 1.82	9.0 ± 3.64	10.4 ± 3.05	***<0.001***	***<0.001***	***0.033***	***0.039***	***0.044***
**Maximal heart rate**	162.6 ± 25.67	161.0 ± 24.87	164.7 ± 24.62	161.9 ± 21.74	157.7 ± 28.08	159.0 ± 31.52	0.702	0.505	0.892	0.395	0.745
**Maximal systolic BP (mmHg)**	203.6 ± 26.01	198.4 ± 23.16	211.2 ± 18.84	206.1 ± 19.82	185.8 ± 31.95	180.3 ± 20.57	0.180	0.228	0.534	***0.010***	***<0.001***
**Maximal diastolic BP (mmHg)**	89.1 ± 12.04	86.4 ± 11.28	90.6 ± 12.35	87.5 ± 11.75	85.5 ± 10.88	84.0 ± 10.04	0.166	0.183	0.654	0.160	0.294

P-values below 0.05 level are marked bold. Values are presented as mean ± SD. 1.M, first measurement; 2.M, second measurement; BP, blood pressure.

The maximal systolic BP achieved during the stress-electrocardiogram test was significantly lower in the DMetS group compared to the NDMetS group at the baseline (185.8 ± 31.95 vs. 211.2 ± 18.84 mmHg, p=0.01) and also following the training program (180.3 ± 20.57 vs. 206.1 ± 19.82 mmHg, p<0.001).

## Discussion

Diabetic cardiovascular autonomic neuropathy (CAN) is defined by the Toronto consensus panel as “the impairment of autonomic control of the cardiovascular system in the setting of diabetes after the exclusion of other causes” (8, 31). CAN is widely recognized as a complication of type 1 and type 2 diabetes mellitus but there is growing evidence that CAN also occurs in patients with obesity, prediabetes or metabolic syndrome, preceding the development of T2DM (7). The population affected by CAN is, therefore, likely to grow as the obesity epidemic continues (16, 32–35). A study in patients with obesity and normal glucose tolerance has demonstrated that increased waist to hip ratio – indicating visceral adiposity – is associated with impaired parasympathetic and sympathetic nervous system control of cardiac autonomic function (36). There is also a significant association between increasing body mass index (BMI) and an increased risk of CAN (14). An early diagnosis of CAN should be established wherever possible since it enables timely implementation of lifestyle and pharmacological interventions aimed at reversing CAN (10, 37). As suggested by the American Diabetes Association (ADA) position statement for diabetic neuropathy, the prevention of CAN is considered the optimal approach for avoiding its adverse consequences (16). Structured lifestyle intervention is recommended already for individuals with impaired fasting glucose or impaired glucose tolerance combined as they are at high risk for developing T2DM, and this approach is likely to reduce CAN (14, 38).

In the present study cardiovascular functional test results in non-diabetic patients with metabolic syndrome were found to be similar to those in patients with diabetic metabolic syndrome. At baseline, only the results of the HRRDB in the diabetic subpopulation showed significant impairment compared to NDMetS patients. This finding confirms an impaired parasympathetic cardiovascular autonomic function at baseline in comparison to the NDMetS group. A three-month training program with remote patient monitoring increased the Valsalva-ratio in the whole group, which provides strong evidence that the condition of the cardiovascular autonomic system can be improved through such a training program. The improvement of the Valsalva-ratio confirms the reinforcement of the parasympathetic tone, which is potentially protective against cardiovascular events. The absence of a significant change in the cardiovascular reflex tests following the training in the DMetS patient group suggests a more seriously impaired adaptation compared to the NDMestS subpopulation of patients with metabolic syndrome.

The systolic blood pressure response to positional change (from lying to standing up) showed a tendency towards improvement in the whole MetSy-population following the training program. It may be speculated that the current study was underpowered to document this change and a study with a larger sample size may be necessary to confirm the potential beneficial effect of physical training on the orthostatic counter regulation.

No further significant differences in autonomic test results could be detected between the two subpopulations at the baseline as well as following training. However, it is worth considering that the duration of diabetes documented in our study population was relatively short, with an average duration of 7.6± 8.12 years, which might explain the absence of a more severe CAN in diabetic individuals with MetS.

A state of sympathetic activation is associated with increased heart rate (39) and appears to promote cardiac and vascular alterations (40, 41), contributing to the development of major complications of hypertension such as arrhythmia, left ventricular hypertrophy and increased arterial stiffening (40–42). It was demonstrated that physical activity such as moderate endurance and aerobic exercise can improve autonomic function as assessed by the heart rate variability (HRV) analysis on the resting ECG (19). Significant improvements in HRV indices were demonstrated in patients with T2DM following a 6-month aerobic exercise training program consisting of 3 training sessions per week. In that study, the subgroup of diabetic patients with definite CAN showed greater improvements in response to a 6-month exercise program compared to the diabetic patients without CAN (21). Michou et al. recently published a study investigating the effects of training on cardiac autonomic neuropathy and metabolic profile in patients with diabetes undergoing hemodialysis. They revealed a positive correlation between peak oxygen uptake and standard deviation of R-R intervals and heart rate after exercise training, which may be an indicator of improved cardiorespiratory efficiency levels affecting cardiac autonomic nervous system function (43). The type and quality of the monitoring and coaching services utilized may be important factors in determining the effectiveness of exercise intervention programs (44). Large, randomized trials in patients with prediabetes and the MetS comprising multifactorial and lifestyle interventions are advocated to establish the effectiveness of different prevention and treatment strategies for patients with prediabetes and CAN (8, 19). Population-based studies demonstrate that there is a higher proportion of deaths - consistent with sudden cardiac death - in CAN patients (19, 45). The mechanisms responsible for increased SCD include an increased incidence of silent myocardial ischaemia and also the prolongation of repolarization possibly leading to severe ventricular arrhythmias and cardiac arrest (46). Thirty years ago Ewing et al. showed in patients with diabetes that the resting QTc correlated with the developmental stage of CAN. They also reported that the resting QTc was longest in individuals who died unexpectedly in follow-up, possibly due to severe cardiac arrhythmias (47).

In the present study no differences were found in the analyzed ECG parameters (including QTc) between the DMetS and NDMetS subgroups at baseline or after a 3-month training period. The resting ECG parameter changes before and after training were significant only in T_peak_-T_end_ at the diabetic subgroup (p=0.043) and at baseline between the DMetS and NDMetS groups (p=0.027). This significant difference following frequency correction with Bazett and Fridericia formulae could not be verified. Furthermore, a three-month long exercise intervention program did not show any effect on the ECG parameters, suggesting its lack of effect on ventricular repolarization.

At the stress-electrocardiogram test in the whole study population, a significant increase of the duration and of the MET score was observed. In addition, a significant improvement was found by the subanalysis of the MET value in the NDMetS and in the DMetS subpopulation. The DMestS patients achieved a significantly lower MET score at baseline and following the training in comparison to the NDMetS patients. Additionally, the DMetS group showed a significantly lower maximal systolic BP during the stress-electrocardiogram test compared to the NDMetS group at baseline and at the second measurement following the training. This finding may be attributed to the stricter and more regular blood pressure control that diabetic patients receive as part of their diabetes care. Regardless of the presence of diabetes in patients with metabolic syndrome, the significant improvement observed in the stress-electrocardiogram and cardiovascular reflex tests following lifestyle-changing exercise therapy underscores its importance and effectiveness as a treatment tool for these patients. The exercise training program applied in the study did not lead to significant changes in ECG repolarization parameters, including QT variability in both diabetic and non-diabetic patients with metabolic syndrome, but the effect of more rigorous intervention should be evaluated.

In summary, the three-month long exercise program improved the Valsalva-ratio and the autonomic score in the MetS patients, confirming the reinforcement of the parasympathetic tone, which is potentially protective against cardiovascular events. The absence of a significant change in the cardiovascular reflex tests following the training in the DMetS patient group suggests a more seriously impaired adaptation compared to the NDMetS subpopulation of patients with metabolic syndrome. The training had some beneficial effect on blood pressure and on the results of the stress-ECG tests in both groups. The exercise training program applied in the study did not lead to significant changes in ECG repolarization parameters, including QT variability in both diabetic and non-diabetic patients with metabolic syndrome, but the effect of more rigorous intervention should be evaluated optimally also in a larger sample size. Further studies need to address the effect of more rigorous exercise protocols with preferably longer duration on the investigated parameters.

## Limitations

In the present study, the home based training was followed by a commercially available system, which is affordable and accessible for widespread utilization. The text message based coaching, included in this system was, however, not suitable for every patient involved, so we used telephone calls and emails to coach the patients. Unfortunately, we were not able to document the regularity and intensity of this coaching activity, which can obviously influence the outcome of the intervention. In subsequent studies, these issues will be addressed. A greater sample size is required to increase the statistical power of the study and allow for a more comprehensive investigation of the relationship between the intensity, extent and type of training and the achieved changes in the neuropathic status. Moreover, further research is warranted to investigate the effect of additional lifestyle interventions including nutrition coaching, on neuropathy.

## Data availability statement

The raw data supporting the conclusions of this article will be made available by the authors, without undue reservation.

## Ethics statement

This study was carried out in accordance with the Declaration of Helsinki (2000) of the World Medical Association and was approved by the Hungarian Medical Research Council (approval No. 50780-2/2017EKU). All subjects have given written informed consent of the study. As a clinical trial our study is also registered at ClinicalTrials.gov (identifier: NCT05146076).

## Author contributions

AV, JÁ, ÉM, AK, MB, AO, PK, AN, TV, IB, CL and IK given substantial contributions to the conception, the methodology, and the design of the manuscript. JÁ given substantial contribution in the patient recruitment, selection, and data collection. AV, ÉM, AK, MB, IB and IK taken part in the investigation, data collection and the interpretation of the results. MS did the statistical analysis. AV participated in writing and drafting the manuscript. AV, ÉM, AN, PK, AM, TV, IB, CL and IK revised the manuscript critically. All authors contributed to the article and approved the submitted version.

## References

[1] Alberti KGMM Eckel RH Grundy SM Zimmet PZ Cleeman JI Donato KA. Harmonizing the metabolic syndrome A joint interim statement of the international diabetes federation task force on epidemiology and prevention. National Heart, Lung, and Blood Institute; American Heart Association; World Heart Federation; International Atherosclerosis Society; and International Association for the Study of Obesity. Circulation (2009) 120(16):1640–5. doi: 10.1161/CIRCULATIONAHA.109.192644 19805654

[2] Jakubiak G Osadnik K Lejawa M Osadnik T Goławski M Lewandowski P. Syndrome most strongly associated with oxidative stress. Antioxidants (2022) 11(1):79. doi: 10.3390/antiox11010079 PMC8773170 35052583

[3] Church TS Thompson AM Katzmarzyk PT Sui X Johannsen N Earnest CP. Metabolic syndrome and diabetes, alone and in combination, as predictors of cardiovascular disease mortality among men. Diabetes Care (2009) 32(7):1289–94. doi: 10.2337/dc08-1871 PMC2699717 19366967

[4] Grundy SM. Metabolic syndrome pandemic. Arterioscler Thromb Vasc Biol (2008) 28:629–36. doi: 10.1161/ATVBAHA.107.151092 18174459

[5] Franco OH Massaro JM Civil J Cobain MR O’Malley B D’Agostino RB. Trajectories of entering the metabolic syndrome: the Framingham Heart Study. Circulation (2009) 120(20):1943–50. doi: 10.1161/CIRCULATIONAHA.109.855817 19884471

[6] Scuteri A Laurent S Cucca F Cockcroft J Cunha PG Mañas LR. Metabolic syndrome across Europe: Different clusters of risk factors. Eur J Prev Cardiol (2015) 22(4):486–91. doi: 10.1177/2047487314525529 PMC4544872 24647805

[7] Alberti KGMM Zimmet P Shaw J. Metabolic syndrome – a new world-wide definition. A Consensus Statement from the International Diabetes Federation. Diabet Med (2006) 23(5):469–80. doi: 10.1111/j.1464-5491.2006.01858.x 16681555

[8] Spallone V. Update on the impact, diagnosis and management of cardiovascular autonomic neuropathy in diabetes: what is defined, what is new, and what is unmet. Diabetes Metab J (2019) 43(1):3–30. doi: 10.4093/dmj.2018.0259 30793549 PMC6387879

[9] Williams SM Eleftheriadou A Alam U Cuthbertson DJ Wilding JPH. Cardiac autonomic neuropathy in obesity, the metabolic syndrome and prediabetes: A narrative review. Diabetes Ther (2019) 10:1995–2021. doi: 10.1007/s13300-019-00693-0 31552598 PMC6848658

[10] Spallone V Ziegler D Freeman R Bernardi L Frontoni S Pop-Busui R. Cardiovascular autonomic neuropathy in diabetes: clinical impact, assessment, diagnosis, and management. Diabetes Metab Res Rev (2011) . 27:639–53. doi: 10.1002/dmrr.1239 21695768

[11] Ewing DJ Boland O Neilson JM Cho CG Clarke BF. Autonomic neuropathy, QTc interval lengthening, and unexpected deaths in male diabetic patients. Diabetologia (1991) 34:182–5. doi: 10.1007/BF00418273 1884890

[12] Brown DW Giles WH Greenlund KJ Valdez R Croft JB. Impaired fasting glucose, diabetes mellitus, and cardiovascular risk factors are associated with prolonged QTc duration. Results from the Third National Health and Nutrition Examination Survey. J Cardiovasc Risk (2001) . 8:227–33. doi: 10.1177/174182670100800407 11551001

[13] Vinik AI Casellini C Parson HK Colberg SR Nevoret ML. Cardiac autonomic neuropathy in diabetes: a predictor of cardiometabolic events. Front Neurosci (2018) . 12:591. doi: 10.3389/fnins.2018.00591 30210276 PMC6119724

[14] Ziegler D Voss A Rathmann W Strom A Perz S Roden M. Increased prevalence of cardiac autonomic dysfunction at different degrees of glucose intolerance in the general population: the KORA S4 survey. Diabetologia (2015) 58(5):1118–28. doi: 10.1007/s00125-015-3534-7 25724570

[15] Laitinen T Lindström J Eriksson J Ilanne-Parikka P Aunola S Keinänen-Kiukaanniemi S. Cardiovascular autonomic dysfunction is associated with central obesity in persons with impaired glucose tolerance. Diabetes Med (2011) 28(6):699–704. doi: 10.1111/j.1464-5491.2011.03278.x 21388444

[16] Pop-Busui R Boulton AJM Feldman EL Bril V Freeman R Malik RA. Diabetic neuropathy: a position statement by the American Diabetes Association. Diabetes Care (2017) 40(1):136–54. doi: 10.2337/dc16-2042 PMC6977405 27999003

[17] Gæde P Vedel P Larsen N Jensen GVH Parving H-H Pedersen O. Multifactorial intervention and cardiovascular disease in patients with type 2 diabetes. New Engl J Med (2003) 348(5):383–93. doi: 10.1056/NEJMoa021778 12556541

[18] Gæde P Lund-Andersen H Parving H-H Pedersen O. Effect of a multifactorial intervention on mortality in type 2 diabetes. New Engl J Med (2008) 358(6):580–91. doi: 10.1056/NEJMoa0706245 18256393

[19] Fisher VL Tahrani AA. Cardiac autonomic neuropathy in patients with diabetes mellitus: current perspectives. Diabetes Metab Syndr Obes (2017) 10:419–34. doi: 10.2147/DMSO.S129797 PMC5638575 29062239

[20] Kluding PM Pasnoor M Singh R Jernigan S Farmer K Rucker J. The effect of exercise on neuropathic symptoms, nerve function, and cutaneous innervation in people with diabetic peripheral neuropathy. J Diabetes Complications (2012) 26(5):424–9. doi: 10.1016/j.jdiacomp.2012.05.007 PMC3436981 22717465

[21] Pagkalos M Koutlianos N Kouidi E Pagkalos E Mandroukas K Deligiannis A. Heart rate variability modifications following exercise training in type 2 diabetic patients with definite cardiac autonomic neuropathy. Br J Sports Med (2008) 42(1):47–54. doi: 10.1136/bjsm.2007.035303 17526623

[22] Máthéné Köteles É Rafael B Korom A Vágvölgyi A Ábrahám JE Domján A. Physiological and psychological effects of a 12-week home-based telemonitored training in metabolic syndrome. Front Cardiovasc Med (2023) 9:1075361. doi: 10.3389/fcvm.2022.1075361 36704473 PMC9871627

[23] Expert Panel on Detection, Evaluation, and Treatment of High Blood Cholesterol in Adults. Executive summary of the third report of the national cholesterol education program (NCEP) expert panel on detection, evaluation, and treatment of high blood cholesterol in adults (Adult treatment panel III). JAMA (2001) 285:2486–97. doi: 10.1001/jama.285.19.2486 11368702

[24] Bull FC Al-Ansari SS Biddle S Borodulin K Buman MP Cardon G. World Health Organization 2020 guidelines on physical activity and sedentary behaviour. Br J Sports Med (2020) 54:1451–62. doi: 10.1136/bjsports-2020-102955 PMC7719906 33239350

[25] Visseren FLJ Mach F Smulders YM Carballo D Koskinas KC Bäck M. 2021 ESC Guidelines on cardiovascular disease prevention in clinical practice: Developed by the Task Force for cardiovascular disease prevention in clinical practice with representatives of the European Society of Cardiology and 12 medical societies with the special contribution of the European Association of Preventive Cardiology (EAPC). Eur Heart J (2021) 42:3227–337. doi: 10.1093/eurheartj/ehab484

[26] Arnett DK Blumenthal RS Albert MA Buroker AB Goldberger ZD Hahn EJ. ACC/AHA guideline on the primary prevention of cardiovascular disease: a report of the American College of Cardiology/American Heart Association Task Force on Clinical Practice Guidelines. Circulation (2019) 140:e596–646. doi: 10.1161/CIR.0000000000000678 PMC7734661 30879355

[27] Ewing DJ Clarke BF. Diagnosis and management of diabetic autonomic neuropathy. BMJ (1982) 285(6346):916–8. doi: 10.1136/bmj.285.6346.916 PMC1500018 6811067

[28] Ewing DJ Martyn CN Young RJ Clarke BF. The value of cardiovascular autonomic function tests: 10 years experience in diabetes. Diabetes Care (1985) 8(5):491–8. doi: 10.2337/diacare.8.5.491 4053936

[29] Baumert M Starc V Porta A. Conventional QT variability measurement vs. template matching techniques: comparison of performance using simulated and real ECG. PloS One (2012) . 7:e41920. doi: 10.1371/journal.pone.0041920 22860030 PMC3408402

[30] Brennan M Palaniswami M Kamen P. Do existing measures of Poincaré plot geometry reflect nonlinear features of heart rate variability? IEEE Trans BioMed Eng (2001) 48:1342–7. doi: 10.1109/10.959330 11686633

[31] Tesfaye S Boulton AJM Dyck PJ Freeman R Horowitz M Kempler P. Diabetic neuropathies: update on definitions, diagnostic criteria, estimation of severity, and treatments. Diabetes Care (2010) 33(10):2285–93. doi: 10.2337/dc10-1303 PMC2945176 20876709

[32] Mokdad AH Serdula MK Dietz WH Bowman BA Marks JS Koplan JP. The spread of the obesity epidemic in the United States, 1991–1998. JAMA (1999) 282(16):1519–22. doi: 10.1001/jama.282.16.1519 10546690

[33] Popkin BM Doak CM. The obesity epidemic is a worldwide phenomenon. Nutr Rev (1998) 56(4):106–14. doi: 10.1111/j.1753-4887.1998.tb01722.x 9584495

[34] Baum P Petroff D Classen J Kiess W Blüher S. Dysfunction of autonomic nervous system in childhood obesity: a cross-sectional study. PloS One (2013) 8(1):e54546. doi: 10.1371/journal.pone.0054546 23358101 PMC3554723

[35] Raelene EM Lenhard MJ. An overview of the effect of weight loss on cardiovascular autonomic function. Curr Diabetes Rev (2007) 3(3):204–11. doi: 10.2174/157339907781368931 18220673

[36] Yadav RL Yadav PK Yadav LK Agrawal K Sah SK Islam MN. Association between obesity and heart rate variability indices: an intuition toward cardiac autonomic alteration—a risk of CVD. Diabetes Metab Syndr Obes (2017) 10:57–64. doi: 10.2147/DMSO.S123935 28255249 PMC5322847

[37] Williams S Raheim SA Khan MI Rubab U Kanagala P Zhao SS. Cardiac autonomic neuropathy in type 1 and 2 diabetes: epidemiology, pathophysiology, and management. Clin Ther (2022) 44(10):1394–416. doi: 10.1016/j.clinthera.2022.09.002 36272822

[38] Gregg EW Boyle JP Thompson TJ Barker LE Albright AL Williamson DF. Modeling the impact of prevention policies on future diabetes prevalence in the United States: 2010–2030. Popul Health Metr (2013) 11(1):18. doi: 10.1186/1478-7954-11-18 24047329 PMC3853008

[39] Valensi P. Autonomic nervous system activity changes in patients with hypertension and overweight: role and therapeutic implications. Cardiovasc Diabetol (2021) 20:170. doi: 10.1186/s12933-021-01356-w 34412646 PMC8375121

[40] Grassi G. Assessment of sympathetic cardiovascular drive in human hypertension: achievements and perspectives. Hypertens Dallas Tex (1979) 2009(54):690–7. doi: 10.1161/hypertensionaha.108.119883 19720958

[41] Grassi G Mark A Esler M. The sympathetic nervous system alterations in human hypertension. Circ Res (2015) 116:976–90. doi: 10.1161/CIRCRESAHA.116.303604 PMC4367954 25767284

[42] Manolis AJ Poulimenos LE Kallistratos MS Gavras I Gavras H. Sympathetic overactivity in hypertension and cardiovascular disease. Curr Vasc Pharmacol (2014) 12:4–15. doi: 10.2174/15701611113119990140 23905597

[43] Michou V Liakopoulos V Roumeliotis S Roumeliotis A Anifanti M Tsamos G. Effects of home-based exercise training on cardiac autonomic neuropathy and metabolic profile in diabetic hemodialysis patients. Life (Basel) (2023) 13(1):232. doi: 10.3390/life13010232 36676181 PMC9866875

[44] Stuckey MI Kiviniemi AM Petrella RJ. Diabetes and technology for increased activity study: the effects of exercise and technology on heart rate variability and metabolic syndrome risk factors. Front Endocrinol (2013) 4:121. doi: 10.3389/fendo.2013.00121 PMC3776944 24065952

[45] Vinik AI Maser RE Mitchell BD Freeman R. Diabetic autonomic neuropathy. Diabetes Care (2003) 26(5):1553–79. doi: 10.2337/diacare.26.5.1553 12716821

[46] Vinik AI Ziegler D. Diabetic cardiovascular autonomic neuropathy. Circulation (2007) 115(3):387–97. doi: 10.1161/CIRCULATIONAHA.106.634949 17242296

[47] Ewing DJ Boland O Neilson JM Cho CG Clarke BF. Autonomic neuropathy, QT interval lengthening, and unexpected deaths in male diabetic patients. Diabetologia (1991) 34(3):182–5. doi: 10.1007/BF00418273 1884890

